# Covid-19 Schutzmaßnahmen in Alten- und Pflegeheimen: zwischen Autonomie und Fürsorge – Ergebnisse einer Interviewstudie

**DOI:** 10.1007/s00481-022-00686-x

**Published:** 2022-03-10

**Authors:** Magdalena Flatscher-Thöni, Elisabeth Holzer, Martin Pallauf, Christiane Kreyer

**Affiliations:** 1 Institut für Public Health, Medical Decision Making und HTA, Department für Public Health, Versorgungsforschung und HTA, UMIT Tirol, Private Universität für Gesundheitswissenschaften, Medizinische Informatik und Technik, EWZ I, 6060 Hall in Tirol, Österreich; 2 Institut für Pflegewissenschaft, Department für Pflegewissenschaft und Gerontologie, UMIT Tirol, Private Universität für Gesundheitswissenschaften, Medizinische Informatik und Technik, Hall in Tirol, Österreich; 3 Institut für Pflegewissenschaft und -praxis, Paracelsus Medizinische Privatuniversität, Salzburg, Österreich

**Keywords:** Ethische Herausforderungen Langzeitpflege, Covid-19-Pandemie, Autonomie, Fürsorge, Pflegepersonen, Ethical issues and challenges, Long-term care, COVID-19 pandemic, Autonomy and care, Care givers

## Abstract

Die vorliegende Interviewstudie untersucht ethische Herausforderungen des Pflegealltags in Einrichtungen der Langzeitpflege aus Sicht der Pflegepersonen während der Covid-19-Pandemie. Durch das explorative, wie auch deskriptive methodische Vorgehen liegen Interviewdaten vor, die vier Themenbereichen zugeordnet werden können, die eine komplexe und teilweise konfliktreiche Arbeits- und Lebenswirklichkeit der Langzeitpflege in der Pandemie aufzeigen. Zum einen werden von den Pflegepersonen die staatlich und institutionell getroffenen Schutzmaßnahmen sowie die daraus resultierenden Einschränkungen der persönlichen Freiheit der Bewohner:innen kritisch reflektiert und damit verbunden der Grad der Selbstbestimmtheit der Bewohner:innen von Alten- und Pflegeheimen in der Pandemie hinterfragt. Zum anderen wird – gegeben der pandemiebedingten Maßnahmen – das pflegerische Handeln im Arbeits- und Lebensort Heim als herausfordernd und die Möglichkeiten pflegerischer Fürsorge als stark eingeschränkt beschrieben. Genannt werden hier als konkrete Herausforderungen veränderte pflegerische Prozesse und Pflegequalität, wie auch eine veränderte Beziehungsqualität zu den Bewohner:innen.

Diese vier Themenbereiche können grundsätzlich den ethischen Prinzipien der Autonomie und Fürsorge zugeordnet werden und präsentieren eine inhaltlich relevante Konkretisierung der prinzipienorientierten ethischen Herausforderungen. In der Zusammenschau und Interpretation der Ergebnisse wird ersichtlich, dass die interviewten Pflegepersonen die Beachtung und Umsetzung des Autonomie-Prinzips im pflegerischen Alltag eng mit dem Fürsorgeprinzip und dem daraus resultierenden Wohltun für die Bewohner:innen verknüpfen.

Unsere Ergebnisse verdeutlichen, dass der in den letzten Jahrzehnten erarbeitete Paradigmenwechsel in der Langzeitpflege durch die Covid-19-Pandemie (zeitweise massiv) geschwächt wurde. Zudem machen die berichteten moralischen Unsicherheiten und Konflikte klar, dass Pflegepersonen Unterstützung hinsichtlich der ethischen Reflexion ihres pflegerischen Handelns benötigen und eine nachhaltige Integration von Ethikberatung in Langzeitpflegeeinrichtungen einen möglichen Lösungsansatz darstellen kann.

## Abstract (en)

**Definition of the problem:**

This interview study investigated ethical issues in long-term care facilities from the perspective of caregivers during the coronavirus disease (COVID-19) pandemic. Due to the explorative as well as descriptive methodological approach, interview data are available and can be assigned to four central topics, which reveal a complex and sometimes conflictual reality of work and life in long-term care during the pandemic. On the one hand, the protective measures taken by the state and the institutions, as well as the resulting restrictions on the personal freedom of the residents are critically reflected and the degree of self-determination of the residents of nursing homes in the pandemic is questioned. On the other hand – given the pandemic-related measures – nursing activities in long-term care facilities as a place of work and life are described as challenging, especially regarding the changed nursing processes and quality of care, as well as due to the changed quality of relationships with the residents.

**Arguments:**

These four topics can basically be assigned to the ethical principles of autonomy and care and present a relevant concretization of the principle-oriented ethical challenges in long-term care during the COVID-19 pandemic. In the synopsis and interpretation of the results, it becomes apparent that the interviewed caregivers closely link the observance and implementation of the autonomy principle in everyday nursing care with the principle of care and the resulting well-being of the residents.

**Conclusions:**

Our results clearly show that the paradigm shift in long-term care, which has been developed over the last few decades, was (temporarily massively) weakened by the COVID-19 pandemic. In addition, the reported moral uncertainties and conflicts make it clear that caregivers need support regarding the ethical reflection of their nursing actions and that a sustainable integration of ethics counselling in long-term care facilities may be a possible approach to a solution.

## Einleitung

Im Frühjahr 2020 verbreitete sich die durch das Coronavirus SARS-CoV‑2 ausgelöste Covid-19-Erkrankung rasant über den gesamten Globus und entwickelte sich zu einer Pandemie, die bis heute massive Auswirkungen auf die Gesundheitsversorgung hat. Ab März 2020 wurden daher in Österreich bundesweit Maßnahmen ergriffen, um die Verbreitung des Virus einzudämmen. Diese Schutzmaßnahmen betrafen in besonderer Weise Langzeitzeitpflegeeinrichtungen, da davon ausgegangen werden musste, dass vor allem ältere Menschen und solche mit Vorerkrankungen ein hohes Risiko für schwere Covid-19-Verläufe haben. Eine der ersten und wesentlichen Schutzmaßnahmen in Österreich war räumliche Distanzierung. Zum Schutz der Risikogruppe der älteren Menschen wurden Besuchsbeschränkungen in Pflegeheimen (bis hin zu Kontakt- und Besuchsverboten) in allen Bundesländern umgesetzt. Zudem wurden systematische Testungen auf eine Covid-19 Infektion bei Bewohner:innen sowie Mitarbeiter:innen der Alten- und Pflegeheime eingeführt, wie auch eine kontinuierliche Beobachtung der SARS-CoV-2-Verbreitung in hoher zeitlicher Dichte verankert (BMSGPK 2020).

In der Zusammenschau der wesentlichen Schutzmaßnahmen in Alten- und Pflegeheimen kann festgehalten werden, dass die Situation in Langzeitpflegeinrichtungen in der Zeit seit Ausbruch der Pandemie im März 2020 primär von der Priorisierung des Infektionsschutzes geprägt war (BMSGPK 2020; AEM 2020). Die Maßnahmen des Infektionsschutzes führten zu einer massiven Veränderung von Strukturen, Abläufen, aber auch zwischenmenschlichen Beziehungen, womit der Lebensort Heim einen kontinuierlichen Wandel erfuhr. Bewohner:innen und Mitarbeitende in Langzeitpflegeeinrichtungen wurden vor besondere Herausforderungen gestellt. Die pandemiebedingten Umstände und Vorgaben, unter denen das Pflegehandeln stattfindet, stehen zum Teil den Werten des Pflegethos diametral entgegen, insbesondere in den Einrichtungen der Langzeitpflege, die in den letzten Jahrzehnten durch einen Paradigmenwechsel geprägt waren (AEM 2020). Dieser Paradigmenwechsel lässt sich vor allem daran festmachen, dass Bewohner:innen von Langzeitpflegeinrichtungen als unabhängige Individuen mit eigenen Rechten wahrgenommen werden und der Gedanke guter Pflege einen Fokus auf die Selbstbestimmung der Gepflegten hat (Aronson und Mahler 2016). Damit einhergehend ist beobachtbar, dass sich auch die rechtlichen Anforderungen an die Qualität und Organisation in der Pflege, wie auch an die Selbstbestimmungsgarantien der Pflegebedürftigen deutlich erhöht haben. Zusammenfassend sind somit die individuellen Bedürfnisse und die Selbstbestimmung der Bewohner:innen zentraler Ansatzpunkt für einen ganzheitlichen, bedarfsgerechten und individuellen Pflegeprozess (Schneekloth und Müller 1998; Schopp et al. 2001).

Dieser Fokus auf das selbstbestimmte Individuum erfordert aus Sicht der Pflegepersonen besondere Sensibilität im Kontext von pflegerischen Entscheidungen und Handlungen (Bockenheimer-Lucius 2007). Professionelle Pflege soll den theoretischen, evidenzbasierten Anforderungen ebenso gerecht werden wie einem objektiv erfassbaren Pflegebedarf und den individuellen Bedürfnissen der Bewohner:innen (Riedel 2013a).

Der pflegerische Alltag führt allerdings oftmals zu Situationen moralischer Unsicherheiten, basierend auf einer Diskrepanz zwischen dem objektiven Pflegebedarf, individuellen Bedürfnissen der Bewohner:innen und einer bestehenden Wertepluralität, die eine ethische Reflexion seitens der Pflegepersonen fordert.

Vor diesem Hintergrund hat die Covid-19-Pandemie das Potenzial, Pflegepersonen sowohl mit kritischen und traumatischen Ereignissen wie auch mit moralischen Herausforderungen zu konfrontieren (Knochel et al. 2020). Pflegepersonen werden in der Pandemie einmal mehr zu moralischen Akteuren, die unmittelbare Verantwortung in der Pandemiebekämpfung übernehmen und damit moralische Unsicherheiten, moralische Konflikte und auch Einschränkungen moralischen Handelns erleben (Knochel et al. 2020; Kuhn und Seidlein 2021). Folgende Beispiele verdeutlichen dies: Eine Bewohnerin will an einem Familientreffen teilnehmen, bei dem nach Angaben der Familie alle Hygieneregeln eingehalten werden, was aber niemand in der Einrichtung kontrollieren kann; Bewohner:innen mit Demenz sind nicht in der Lage, Hygieneregeln zu verstehen, und halten sich daher nicht daran; sollen in diesen Fällen freiheitsbeschränkende Maßnahmen eingesetzt werden? (Ethikkommission der Pflegekammer Niedersachsen 2021).

Derartige Handlungs- und Entscheidungssituationen machen seitens der Pflegepersonen umfassende Abwägungen unterschiedlicher moralischer Güter, die einerseits auf den Bedürfnissen der pflegebedürftigen Personen beruhen und sich andererseits aus Pflichten, die sich aus der Einhaltung der medizinethischen Prinzipien (Respekt vor der Selbstbestimmung, Wohltun, Schadensvermeidung und Gerechtigkeit, Beauchamp und Childress 2019) ergeben, notwendig. Zudem gilt es die rechtlichen Rahmenbedingungen im Umgang mit Bewohner:innen einzuhalten.

Vor diesem Hintergrund erscheint es bedeutsam zu verstehen, wie Pflegpersonen die mit ihrer pflegerischen Tätigkeit einhergehenden ethischen Herausforderungen, also jene Situationen, in denen nicht unmittelbar klar ist, was das richtige oder bessere oder wenigstens weniger schlechte Verhalten ist, während der COVID-19-Pandemie wahrnehmen.

Während in der wissenschaftlichen Literatur das Thema ethische Herausforderungen in der Langzeitpflege sowohl theoretisch wie auch empirisch schon seit längerer Zeit ausführlich bearbeitet wird (u. a. Elander et al. 1993; Mattiasson und Andersson 1995; Suhonen et al. 2019; Podgorica et al. 2020), ist die aktuelle Studienlage zu ethischen Herausforderungen von Pflegepersonen während der Covid-19-Pandemie im stetigen Aufbau begriffen (siehe unter anderem Morley et al. 2020a; Sahebi et al. 2020; Gebreheat und Teame 2021; Hödl et al. 2021; Parekh de Campos und Daniels 2021; Sperling 2021). Fokussiert man in diesem Kontext den deutschsprachigen Raum, so ist feststellbar, dass theoretische Abhandlungen, wie auch Stellungnahmen unterschiedlicher Institutionen pflegeethische Positionierungen und Reflexionen veröffentlichen. Zu nennen sind hier neben der Arbeit der nationalen Ethikräte zum Thema Covid-19^1^ vor allem die Publikationen von Fachgesellschaften, wie beispielsweise jene der Akademie für Ethik in der Medizin (z. B. AEM 2020 oder Knochel et al. 2020). Die empirische Verortung des Themas ist ebenso gekennzeichnet durch den dynamischen Verlauf der Pandemie und untersucht neben der Arbeitsbelastung auch psychische Belastungen von Pflegenden, sowie auch Moral Distress im Kontext pflegerischen Handelns (Begerow et al. 2020; Begerow und Gaidys 2020; Bohlken et al. 2020; Morley et al. 2020b; Petzold et al. 2020; Robert et al. 2020; Sperling 2021; Donkers et al. 2021; Ness et al. 2021).

In der Zusammenschau dieser empirischen Literatur zeigt sich, dass bis dato der Fokus vor allem auf die Intensivpflege in Krankenanstalten und die damit verbundenen (organisations-) ethischen Herausforderungen gelegt wurde. Die empirische Untersuchung der ethischen Herausforderungen in der stationären Langzeitpflege während der Covid-19-Pandemie aus Sicht der Pflegepersonen liegt allerdings – soweit bekannt – für den deutschsprachigen Raum und den damit verbundenen Charakteristika der entsprechenden Gesundheitssysteme noch nicht vor.

An diesem Punkt setzt die vorliegende qualitative Studie an und geht der Frage nach, welche ethischen Herausforderungen von Pflegepersonen in der stationären Langzeitpflege während der Covid-19-Pandemie wahrgenommen wurden.

## Methode

Die vorliegende empirische Studie ist sowohl explorativ als auch deskriptiv angelegt. Da sich die leitende Fragestellung der Studie auf die subjektiven Wahrnehmungen und Erfahrungen ethischer Herausforderungen von Pflegepersonen während der Covid-19-Pandemie konzentriert, rücken Phänomene ins Zentrum des Forschungsinteresses, die sich versprachlichen lassen, weshalb ein qualitativer Ansatz gewählt wurde.

Die Entscheidung für einen qualitativen Zugang erlaubt es, ein vertieftes Verständnis sozialer Phänomene zu erlangen, indem das Erleben und Handeln der betroffenen Akteure in den Blick genommen wird (Flick 2017; Polit und Beck 2009).

### Datenerhebung

Die Datenerhebung erfolgte anhand problemzentrierter Leitfadeninterviews, in denen das Vorwissen der Forschenden als heuristisch-analytischer Rahmen für Frageideen im Dialog zwischen Interviewenden und Befragten dient, wobei gleichzeitig Narrationen angeregt werden (Witzel 2000). Durchgeführt wurden die Interviews von vier diplomierten Gesundheits- und Krankenpflegepersonen mit langjähriger Erfahrung sowohl in der pflegerischen Versorgung als auch in der qualitativen Pflegeforschung. Die Interviews wurden durch einen Leitfaden strukturiert, der literaturgestützt erarbeitet wurde und offene, erzählgenerierende Fragen enthielt (Helfferich 2011). Die Themenbereiche waren: (1) Ausbildung und Motivation in der Langzeitpflege zu arbeiten, (2) ethische Herausforderungen im pflegerischen Alltag während der Covid-19-Pandemie, (3) Lösungsansätze/-strategien für ethisch herausfordernde Situationen während der Covid-19-Pandemie und (4) Arbeitsbelastung während der Covid-19-Pandemie.

Interviewpartner:innen wurden, nach institutioneller Einwilligung, in Tiroler Alten- und Pflegeheimen rekrutiert. Eingeschlossen wurden Pflegepersonen, die im pflegerischen Alltag tätig sind, um ethische Herausforderungen, die in der direkten Pflege entstanden sind, zu beleuchten. Dies sind Fachpflegepersonen (in Österreich: Diplomierte Gesundheits- und Krankenpflegepersonen), Pflege(fach)assistent:innen, Sozialbetreuer:innen sowie auch Pflegedienstleitungen. Mittels eines selektiven Samplingverfahrens (Flick et al. 2017) wurde eine maximale Variationsbreite demografischer, beruflicher und regionaler Merkmale angestrebt, um die relevanten Erfahrungen und Sichtweisen im Feld breit erfassen zu können.

Mit Personen, die Interesse an einem Interview zeigten, wurde – soweit dies die Covid-19-Schutzmaßnahmen zuließen – ein Face-to-Face-Interview geführt; in allen anderen Fällen wurde das Interview via Telefon oder Videokonferenz durchgeführt. Die Teilnehmenden erhielten im Vorfeld des Interviews ein Informationsschreiben zur Studie (inklusive der relevanten Informationen zur Anonymität der Datenerhebung und der Kontaktmöglichkeit zu den Forscher:innen), sowie die Einverständniserklärung. Nach nochmaliger mündlicher Aufklärung zu Beginn der Interviews erteilten sie ihre Zustimmung zur Aufnahme und Auswertung der Daten. Besondere Sorgfalt wurde daraufgelegt, die Anonymität der Teilnehmer:innen und der beteiligten Einrichtungen zu sichern. Es wurden schließlich fünf persönliche, sechs telefonische und sieben Interviews via Videokonferenz durchgeführt, die zwischen 20 und 80 min dauerten.

Der Zeitraum der Datenerhebung (Oktober 2020 bis Februar 2021) war von physischer Distanzierung, zeitweisen Kontaktsperren, wie auch sozialen und wirtschaftlichen Einschränkungen geprägt. Konkret kam es in diesem Zeitraum in Österreich zu zwei harten Lockdowns, die sowohl Ausgangssperren, Maskenpflicht, beschränkte Besuchsmöglichkeiten, wie auch Besuchsverbote in Alten- und Pflegeheimen beinhalteten.

### Datenanalyse

Die Daten wurden anhand der inhaltlich strukturierenden qualitativen Inhaltsanalyse nach Kuckartz (2018) ausgewertet, die das Ziel hat, Textmaterial systematisch und regelgeleitet zu analysieren. Die Datenorganisation und Analyse erfolgte unter Verwendung der Software MAXQDA 2020. Nach der Transkription der Interviews und der initiierenden Textarbeit wurden von den Autor:innen thematische Hauptkategorien entwickelt, die einerseits aus dem Interviewleitfaden abgeleitet und andererseits durch Themen ergänzt wurden, die sich im Material fanden. Das gesamte Material wurde durch zwei Forscherinnen codiert. Im nächsten Schritt wurden für jede Hauptkategorie induktiv Subkategorien gebildet und das Material erneut codiert. Schließlich wurden die Hauptkategorien kategorienbasiert analysiert und verdichtet, sowie nach Zusammenhängen zwischen den Kategorien gesucht. Alle Auswertungsschritte wurden im Autor:innenteam diskutiert bis Konsens erzielt wurde.

Das Forschungsvorhaben wurde vor Beginn der Untersuchung dem zuständigen universitären Ethikgremium (RCSEQ – Research Committee for Scientific Ethical Questions, UMIT Tirol), das wissenschaftliche Arbeiten vor Durchführung auf wissenschaftlich-ethische Kriterien prüft, gemeldet und als unbedenklich eingestuft (Aktenzeichen 2802).

## Ergebnisse

Die im Rahmen dieses Beitrags präsentierten Ergebnisse beruhen auf 18 Interviews, die im Erhebungszeitraum Oktober 2020 bis Februar 2021 im österreichischen Bundesland Tirol geführt wurden.^2^ Die 15 Frauen und drei Männer waren zwischen 25 und 57 Jahre (Mittelwert: 43 Jahre) alt. Drei hatten eine Ausbildung als Pflege(fach)assistent:in, zwölf als Diplomierte Gesundheits- und Krankenpflegeperson sowie drei einen Bachelor- oder Masterabschluss, zusätzlich zu ihrer Ausbildung als Diplomierte Gesundheits- und Krankenpflegeperson. Sechs der Teilnehmenden waren ausschließlich in der direkten Pflege tätig, sieben zusätzlich als Wohnbereichsleitung, fünf hatten die Funktion Pflegedienstleitung oder deren Stellvertretung inne. 15 der Pflegepersonen waren in ländlichen und drei in städtischen Heimen beschäftigt. Zehn der interviewten Personen arbeiteten Vollzeit in der Pflegeeinrichtung, acht in Teilzeitpositionen. Hinsichtlich der Arbeitserfahrung in der Langzeitpflege deckte die Studienpopulation eine große Bandbreite ab (0–5 Jahre = 5, 6–10 Jahre = 4, 11–15 Jahre = 5, mehr als 15 Jahre = 4). Zusammenfassend weist das Sample damit ein hohes Maß an Heterogenität auf.

Aus den Interviewdaten wurden vier Kategorien identifiziert, die nachfolgend präsentiert und mit wörtlichen Zitaten aus den Narrativen der Pflegepersonen untermauert werden.

### Persönliche Freiheit „… du hast gemerkt, das macht was mit den Leuten.“

Covid-19-Schutzmaßnahmen^3^ knüpfen auch in der stationären Langzeitpflege an Einschränkungen der persönlichen Freiheit an. Durch die gesundheitsbehördlichen Beschränkungen der persönlichen Freiheit der Bewohner:innen in Pflege- und Betreuungseinrichtungen wurden Alten- und Pflegeheime (zeitweise) von der Außenwelt isoliert; gleichzeitig kam es aber auch nach positiver Testung auf Covid-19 zu einer Absonderung (Isolierung) bestimmter Personen und allfälliger Kontaktpersonen auf individueller Ebene (individuelle Beschränkungen, die in der Regel mit behördlichem Bescheid angeordnet wurden).

In der Praxis führten diese Maßnahmen dazu, dass nicht infizierte Bewohner:innen lange Zeit in Zimmerquarantäne bleiben mussten – vom Gemeinschaftsleben isoliert – und infizierte Personen auf Isolierstationen dagegen in der Gruppe der Erkrankten in einer Gemeinschaft leben konnten und versorgt wurden. „Zimmerquarantäne hatten wir durchgehend […], da haben es die Menschen, die infiziert waren auf der Isolierstation schöner gehabt, […] weil sie miteinander in diesem abgesperrten Bereich kommunizieren konnten. […] die haben mehr Hausgemeinschaft und Sozialkontakte gehabt, die Kranken mehr als die Gesunden.“ (Interview P).

Die Bewegungsbeschränkungen im Sinne der Zimmerquarantäne der einzelnen Bewohner:innen brachten für die Pflegepersonen unterschiedliche Herausforderungen mit sich. Vor allem die damit verbundene soziale Isolation sowohl durch das Besuchsverbot von außen, wie auch die durch die internen Kontaktbeschränkungen nur mehr marginal vorhandene Gemeinschaft in den Einrichtungen hatten zur Folge, dass Bewohner:innen „[…] körperlich und geistig total abbauten“ (Interview C). Pflegepersonen beschrieben diese Kontaktbeschränkungen mit „[…] man [einfach] sieht, wie sie leiden, […] das macht was mit den Leuten.“ (Interview M) und „[…] wir sind die einzigen Bezugspersonen.“ (Interview M). „[…] Also das Schlimmste ist der Besuchsentzug und bei den K1 Personen^4^ [Kontaktpersonen] die Einzelquarantäne.“ (Interview R). Zusammenfassend stellten die Pflegepersonen fest, dass die Isolation der Bewohner:innen zu Angst und Unsicherheiten führte, die ein hohes Maß an psychischer Belastungen mit sich brachte. Verstärkt wurden diese Herausforderungen bei Menschen mit kognitiven Einschränkungen bzw. Veränderungen. „[…] Wir sind durchgehend beschäftigt, die Leute in die Zimmer zurückzubringen, […]. Die sind dement, die wollen raus, die verstehen das nicht. Und dann stürzen sie natürlich […], weil sie im Zimmer aufstehen, da hat man sie auch nicht unter Kontrolle […], sonst sitzen sie in der Wohnküche.“ (Interview L). Eine Pflegeperson thematisiert freiheitsbeschränkende Maßnahmen mit „Ein Bewohner mit Demenz, der großen Bewegungsdrang hat, wurde zehn Tage in Zimmerquarantäne gehalten. Um zu verhindern, dass er herumgeht, wurde er 10 Tage nicht aus dem Bett mobilisiert und mit Gitter gesichert. Der Bewohner ist mehrmals gestürzt, als er versucht hat, das Bett zu verlassen. Ein anderer Bewohner wurde medikamentös ruhiggestellt.“ (Interview L).

Viele Pflegepersonen reagierten auf diese Situationen im Sinne eines moralischen Konflikts. In den Interviews wurde ein grundsätzliches Verständnis für die rechtlich bestimmten Schutzmaßnahmen und die daraus resultierenden Konsequenzen für ihren pflegerischen Alltag formuliert, allerdings nahmen sie auch auf die entsprechenden Belastungen, also Leid, Trauer und Unverständnis der Bewohner:innen Bezug. „[…] wie gesagt, wenn wer positiv ist und man muss ihn isolieren, kein Thema. Aber so vorsorglich gleich einmal alle isolieren […].“ (Interview C).

Belastungen wurden auch seitens der Pflegepersonen berichtet, vor allem in Fällen wiederholter Quarantänen von Bewohner:innen. Eine Pflegeperson konkretisierte: „Und irgendwann, du kannst nicht mehr, wir haben gesagt, es geht nicht, das kann es nicht sein, dass die jetzt schon wieder zehn Tage eingesperrt wird.“ (Interview M).

Basierend auf diesen Konflikten wurden durch Pflegende Strategien entwickelt, die den rechtlichen Rahmen der Pandemiebekämpfung weit interpretiert haben, um den Bewohner:innen ein Mindestmaß an sozialem Austausch ermöglichen zu können. „Also das mit im Zimmer essen und nicht raus, da hätte ich auch ein massives Problem gehabt, wenn wir das so machen hätten müssen. Das wäre ethisch für mich nicht vertretbar. […]“ (Interview C).

Einzelne Interviews weisen zudem darauf hin, dass Pflegepersonen auch autonom darüber entschieden haben, ob Bewohner:innen als Kontaktpersonen der Behörde gemeldet wurden. Die damit einhergehende Verantwortungsübernahme erfolgte aus einem fürsorglichen Schutzgedanken den Bewohner:innen gegenüber, führte aber gleichzeitig zu einem moralischen Konflikt: „[…] was du zum Schutz für jemanden machst kann auch zu einem schlechten Gewissen führen.“ (Interview R).

Pflegepersonen reflektierten das Thema physische Distanzierung durch Bewegungsbeschränkungen (Besuchs- und Kontaktverbot) grundsätzlich kritisch. „Und das war gerade für uns Pflegepersonen eine riesige Herausforderung, die Leute auch psychisch zu stabilisieren und […] dieser Lockdown hat den Leuten das Persönliche weggenommen […]“ (Interview D). Auch wurde vermehrt die zeitliche Dimension der Pandemie angesprochen und hier vor allem das Unwissen über die Dauer bzw. das mögliche Ende. „Die ältere Generation, […] also 80 plus, sind der Meinung gewesen, ihnen wäre lieber, dass die Angehörigen hereindürfen […] da man ja nicht weiß, wie lange Corona noch dauert […]“ (Interview A). Ebenso wurde die verbleibende Lebenszeit mit der damit verbundenen Lebensqualität in Verbindung gesetzt, so wurde von Bewohner:innen berichtet, die eine explizite Abwägung vorgenommen haben: „[…] dann lebe ich vielleicht zwei bis drei Monate länger und habe aber keine Lebensqualität mehr und das stört mich.“ (Interview A).

Auch wurde in diesem Kontext einmal mehr die Selbstbestimmung der Bewohner:innen betont: „Aber viele haben gesagt […] uns fragt gar niemand, ob wir das eigentlich wollen. […] kann ich das nicht mit 94 bestimmen, ob ich vielleicht lieber an dem Virus sterbe und meine Kinder sehe, als wie ich werde da eingesperrt über Wochen und es passiert nichts mehr.“ (Interview H).

### Selbstbestimmtheit der Bewohner:innen „… und dann hat er nein gesagt.“

Autonome Entscheidungen von Bewohner:innen in Langzeitpflegeeinrichtungen wurden in der Literatur vor der Covid-19-Pandemie bereits ausführlich diskutiert und vor allem auf die zentralen Aspekte Selbstbestimmung und Handlungsfähigkeit zurückgeführt (Wulff und Dräger 2012; Wulff 2013). Diese selbstbestimmte Entscheidungsfindung durch Bewohner:innen führte im Kontext der Schutzmaßnahmen, vor allem von Testungen und der Corona-Impfung, zu neuen Herausforderungen. „[…] es soll ja nicht so sein, dass wir über sie bestimmen. Sie sollen ja selbst sagen können, was sie wollen. Wir können es ja auch sagen […].“ (Interview O).

„Was tun wir, wenn sich jemand nicht testen lassen will?“ (Interview N). Fragen wie diese beschäftigten Pflegepersonen im Kontext der (teilweise behördlich angeordneten) Covid-19-Testungen. Wenngleich wenige Pflegepersonen berichteten, dass Bewohner:innen „nein gesagt“ haben, führten Testungen, die im Verlauf der Pandemie vor allem von Pflegepersonal durchgeführt wurden, zu „ethischen Schwierigkeiten“ (Interview G). „Und da haben wir auch oft die Bewohner niederhalten müssen, so grausig das war, dass wir zu einem Abstrich [Rachen- oder Nasen] gekommen sind. […] Und die haben sich nicht ausgekannt, wir haben die Schutzmasken. Ich habe mir oft gedacht, die werden sich gedacht haben, die sind wieder im Krieg.“ (Interview B). „Wir haben eine Bewohnerin, wir mussten sie zu viert festhalten, dass wir diesen Nasenabstrich machen konnten und […] ich tu das nicht mehr […].“ (Interview Q). Pflegepersonen nahmen diese zwanghafte Abnahme von Testungen sehr konfliktbehaftet wahr und sahen darin Grenzüberschreitungen, die für sie nicht mehr tragbar waren. „Was sollen wir denn machen, wie sollen wir die Leute testen, wenn sie nicht herhalten? Dann haben wir auch im Team Probleme bekommen: ‚wie kann man nur so brutal sein und die festhalten‘ […].“ (Interview Q). Ebenso wurde berichtet, dass Testungen einen negativen Einfluss auf die Beziehung zu den Bewohner:innen hatten, vor allem im Hinblick auf das notwendige Vertrauensverhältnis im Umgang mit (dementen) Bewohner: innen (Interview R).

Eine Pflegeperson fasste den Grenzgang, der von ihrer Berufsgruppe verlangt wurde und zu einem moralischen Dilemma führte, wie folgt zusammen: „[…] ich kann ihr zehn Mal erklären, ich brauche das, sie verstehen es nicht. Und da denke ich mir jedes Mal, wenn das die Heimanwältin sehen würde oder die Bewohnervertretung, – ein absolutes No-Go. Und dass ich das dann auch noch machen muss auch noch, damit ich einfach diesen […] Bestimmungen entspreche. Ja was macht das mit dir, wenn du das tun musst? […]“ (Interview R).

Die Impfentscheidungen von Bewohner:innen wurden von Pflegepersonen unterschiedlich beschrieben. Bei gegebener Entscheidungsfähigkeit (in Deutschland: „Einwilligungsfähigkeit“), nahmen die Bewohner:innen ihre Selbstbestimmung im Sinne einer autonomen Entscheidungsfindung wahr. „Aber es sind einige Bewohner, die […] sagen, […] nein ich lasse mich nicht impfen, ja was sollen wir dann, wir können Sie ja nicht vergewaltigen […]. Die Bewohner, die das selbst nicht mehr so sagen können, die sind wohl geimpft worden.“ (Interview O). Pflegende konkretisierten diese fallweise Abkehr von der Selbstbestimmung kritisch: „[…] ich bin jetzt schon so lang in der Pflege, ich weiß wirklich noch, in meiner Anfangszeit war es ganz normal, dass wir Leute an das Bett gefesselt haben, […] Gott sei Dank ist die Pflege in der Hinsicht sehr gewachsen und hat sich sehr verändert. […] Aber jetzt gebe ich die [Impf-]Spritzen und ich mache die Tests, da vergewaltige ich sie ja und das ist völlig egal, oder? […]“ (Interview O).

### Veränderte Beziehungsqualität „… wir sind die einzigen Bezugspersonen.“

Die Priorisierung des Infektionsschutzes in Langzeitpflegeeinrichtungen hatte einen erheblichen Einfluss auf den Arbeits- wie auch Lebensort Heim. Es kam hierbei zu massiven Veränderungen der Prozesse sowie Tagesstruktur und -gestaltung. Vor allem Pflegepersonen nahmen die damit einhergehende Dysfunktionalität des Arbeits- und Lebensortes wahr: „[…] es hat einfach nicht mehr funktioniert […]“ (Interview D). Oder auch: „[…] ihr Rhythmus, den sie seit Jahren haben, fehlt […]“ (Interview M).

Ansatzpunkte für diese Wahrnehmung, die eigene (relationale) Funktion nicht erfüllen und ausfüllen zu können, zeichneten sich in den Interviews auf unterschiedlichen Ebenen ab. Pflegepersonen berichteten in den Interviews über eine veränderte Nähe zu den Bewohner:innen, die sich sowohl auf einer emotionalen, wie auch einer physischen Ebene niederschlug. Zum einen führten die Kontaktbeschränkungen zu keinen Besuchen von außen, sodass die Pflegepersonen „[…] eigentlich die einzigen Kontaktpersonen in der Zeit […]“ (Interview F) waren und damit verbunden viel Beziehungsarbeit leisten hätten müssen. Vor allem weil sich viele Bewohner:innen „[…] alleine fühlten […]“ (Interview M) und das Klima in den Heimen durch (psychische) Unruhe und Unsicherheit geprägt war.

Diesem erhöhten Bedarf an emotionaler Nähe – im Sinn einer verstärkten Beziehungsarbeit – standen allerdings die behördlichen Hygienevorgaben im Umgang mit infizierten Bewohner:innen (Kontaktpersonen), wie auch mit gesunden Bewohner:innen entgegen. „[…] man soll schon versuchen Abstand zu halten […] aber es ist ethisch für mich schwer vertretbar […].“ (Interview F). Dieses Spannungsfeld wurde verstärkt, durch das Wissen der Pflegperson, dass die Bewohner:innen „[…] wenig oder gar keine Zuneigung bekommen […]“ (Interview F) und sie zeitweise „[…] die einzigen Bezugspersonen […]“ (Interview M) waren. Zudem berichteten Pflegepersonen von der emotionalen Belastung der Bewohner:innen als Konsequenz der Isolierung „[…] sie weinen oft oder halten uns fest […].“ (Interview M).

### Veränderte Pflegequalität „… ich habe das Problem, dass ich meine Pflege nicht so machen kann, wie ich sollte.“

Das in der Langzeitpflege etablierte Konzept des ganzheitlichen, bedarfsgerechten und individuellen Pflegeprozesses betrachtet Bewohner:innen nicht als reine Fürsorgesubjekte, sondern nimmt sie als autonome Individuen mit eigenen Rechten wahr und baut somit auf eine vernetzte Sicht des gesamten Menschen auf. Dieser ganzheitliche Ansatz wurde durch die Covid-19-Schutzmaßnahmen geschwächt. Als Ausgangspunkt für die (zeitweise) Abkehr von diesem Ansatz konnte die aufgrund der Hygienemaßnahmen vorgegebene Kontakteinschränkung zwischen Pflegepersonen und Bewohner:innen und die daraus resultierende zeitlich stark limitierte Pflegearbeit identifiziert werden. Eine Pflegeperson konkretisierte: „[…] wir konnten den integrativen Pflegeprozess fast nicht mehr umsetzen. Die Autonomie des Menschen zu stärken, Biografie Arbeit zu machen […], die Umsetzung unseres Pflegekonzepts funktioniert nicht mehr und das ist schade und traurig […].“ (Interview D).

Daraus resultierende moralische Konfliktsituationen wurden besonders im Umgang mit Bewohner:innen mit kognitiven Einschränkungen betont „[…] du kannst nicht in zehn Minuten einen dementen Menschen pflegerisch versorgen, der ist im Anschluss total verwirrt. Er hat dich nicht erkannt, er hat nicht gewusst, was du machst, und zusätzlich hast du schauen müssen, dass du den zeitlichen Vorgaben entsprichst […].“ (Interview M).

Als zusätzliche Herausforderung für Pflegepersonen wurde das Tragen eines Mund-Nasen-Schutzes, vor allem in der ersten Zeit der Pandemie, genannt. Zum einen, da die Bewohner:innen durch die Masken verunsichert und verängstigt waren. Zum anderen weil der Mund-Nasen-Schutz zu einer sehr eingeschränkten Kommunikation führte, da die Bewohner:innen nicht mehr von den Lippen lesen konnten und die Pflegepersonen nur schwer verstanden haben (Interviews A; E; P; Q).

Eine weitere Ebene der Einschränkung war der stark veränderte Tagesablauf, der sich zum einen daraus ergab, dass externe Personen wie Physio- oder Ergotherapeut:innen entsprechende Aktivitäten nicht mehr anbieten konnten, und zum anderen Unterstützung durch Zivildiener oder ähnliches nicht mehr möglich war. „[…] [D]ie Herausforderung war auch, dass man versucht hat den Tagesablauf so zu halten, wie er war. Man hat versucht Aktivitäten anzubieten, spazieren zu gehen etc. […]. Aber in dem Ausmaß unseres ursprünglichen Pflegekonzepts kann man es einfach nicht leisten.“ (Interview F).

Eine Pflegeperson fasste die Situation folgendermaßen zusammen: „[…] Es bräuchte mehr Personal, damit man mit gutem Gewissen rausgehen kann, also die Pflegequalität adäquat ist.“ (Interview L).

## Diskussion

Durch das explorative, wie auch deskriptive methodische Vorgehen liegen Interviewdaten vor, die vier Themenbereichen zugeordnet werden können, die eine komplexe und teilweise konfliktreiche Arbeits- und Lebenswirklichkeit der Langzeitpflege in der Pandemie aufzeigen. Zum einen werden die staatlich und institutionell getroffenen Schutzmaßnahmen, sowie die daraus resultierenden Einschränkungen der persönlichen Freiheit der Bewohner:innen kritisch reflektiert und damit verbunden der Grad der Selbstbestimmtheit der Bewohner:innen von Alten- und Pflegeheimen in der Pandemie hinterfragt. Zum anderen wird – gegeben der pandemiebedingten Maßnahmen – das pflegerische Handeln im Arbeits- und Lebensort Heim als herausfordernd beschrieben, vor allem hinsichtlich der veränderten pflegerischen Prozesse und Pflegequalität, wie auch aufgrund der veränderten Beziehungsqualität zu den Bewohner:innen.

Unsere Ergebnisse verdeutlichen, dass der in den letzten Jahrzehnten erarbeitete Paradigmenwechsel in der Langzeitpflege durch die Covid-19-Pandemie, vor allem durch die Priorisierung des Infektionsschutzes und die daraus resultierenden Konsequenzen für Bewohner:innen und Pflegepersonen, (zumindest zeitweise massiv) geschwächt wurde (AEM 2020; Giese 2020; Kohlen et al. 2019). So wurden zum einen die individuellen Bedürfnisse und zum anderen die Selbstbestimmtheit der Bewohner:innen von Langzeitpflegeeinrichtungen, die zentrale Ansatzpunkte für einen ganzheitlichen, bedarfsgerechten und individuellen Pflegeprozess (Schneekloth und Müller 1998; Schopp et al. 2001) darstellen, durch pandemiebedingte Maßnahmen relativiert.

Gleichzeitig ermöglichen die vier Themenbereiche auch eine inhaltliche Konkretisierung der prinzipienethischen Ansatzpunkte der Autonomie und Fürsorge (Monteverde 2012; Riedel 2013b; Aronson und Mahler 2016; Beauchamp und Childress 2019). Die vorliegenden Studienergebnisse zeichnen ein konkretes Bild des pflegerischen Alltags, der sich in Zeiten der Pandemie vor allem zwischen der Verwirklichung der Selbstbestimmheit der Bewohner:innen und dem Fokus auf das geistige, körperliche, seelische und soziale Wohl der Bewohner:innen bewegt. Die Fähigkeit, ein selbstbestimmtes Leben als Bewohner:in einer Langzeitpflegeeinrichtung führen zu können, hängt von vielen Faktoren ab. Die grundlegendsten sind die Fähigkeit des Menschen und seine Möglichkeiten, Präferenzen sowie Ziele und damit sein eigenes Wertesystem selbst gestalten zu können. Demzufolge bedeutet die Verwirklichung des Autonomie-Prinzips in der Langzeitpflege, dass die zu betreuende Person als Selbstzweck geachtet wird, ihre Selbstbestimmungsrechte anerkannt werden und sie in jeder Lage und in jedem Zustand bestmöglich gefördert wird (Wulff 2013; Schweda et al. 2018).

Die daraus ableitbare Verantwortlichkeit der Pflegepersonen ist der Ansatzpunkt für ethische Herausforderungen, über die in den vorliegenden Interviews berichtet wurde. Während die Corona-bedingten freiheitsbeschränkenden Maßnahmen in ihrer behördlicher Anordnung jede:n Bewohner:in individuell in der Umsetzung trafen, waren Pflegepersonen gleichzeitig auch verpflichtet, an dieser Umsetzung mitzuwirken (Zierl 2020). Die aus der Umsetzung der Maßnahmen resultierende soziale Isolation der Bewohner:innen, als auch die tatsächliche räumliche Einschränkung, führten beim Pflegepersonal wiederum zu moralischen Unsicherheiten und Konflikten. Erweitert wurde die kritische Reflexion dieser Einschränkung der Selbstbestimmheit durch die Erfahrungen hinsichtlich autonomer Entscheidungen von Bewohner:innen im Kontext der Covid-19-Testungen und -Impfungen. Auch hier zeigten sich Pflegepersonen besorgt angesichts der Eingriffe in die selbstbestimmte Entscheidungsfindung der Bewohner:innen.

In diesen Phasen von Unsicherheit und Konflikten verdichtete sich die Rolle der Pflegepersonen als moralische Akteure, da sie in den jeweiligen Situationen durch individuelle Verantwortungsübernahme, beispielsweise das Schaffen gemeinsamer Lebens- und Essräume, das Akzeptieren einer Testverweigerung oder der Impfverweigerung, den Versuch unternommen haben, die Selbstbestimmung der Bewohner:innen nur soweit als unbedingt notwendig einzuschränken.

Diese fürsorgliche Unterstützung der Selbstbestimmung leitet bereits auf den zweiten Bereich ethischer Herausforderungen über. Im Fokus steht hier der Umgang mit dem körperlichen, geistigen, seelischen und sozialen Wohl der Bewohner:innen (Wulff 2013; Schweda et al. 2018). Dieser Respekt der persönlichen Entfaltungsmöglichkeiten und die daraus resultierende Fürsorge für die Bewohner:innen, im Sinne eines ganzheitlichen Pflegeprozesses, wurde gemäß den Berichten der Pflegepersonen durch das Einhalten der Corona-Maßnahmen stark eingeschränkt.

So wurde zum einen von einer veränderten Beziehungsqualität zwischen Bewohner:innen und Pflegepersonen berichtet, die zu moralischen Konflikten führte. Das behördlich geforderte Mitwirken der Pflegepersonen an Covid-19-Testungen führte bei den Bewohner:innen zu einem Misstrauen den Pflegepersonen gegenüber und damit verbunden zu einer veränderten Vertrauensgrundlage. Das Wissen und Erfahren der sozialen Isolation der Bewohner:innen verstärkte bei Pflegepersonen aber auch das Bedürfnis, für die Bewohner:innen da zu sein, Ersatzmensch zu sein und die mangelnden bzw. inexistenten sozialen Kontakte zu kompensieren.

Zum anderen war es den Pflegenden nicht mehr möglich, eine zufriedenstellende Pflegequalität aufrecht zu erhalten, sodass die pandemiebedingte Arbeitswirklichkeit dem (individuellen) Pflegeethos diametral entgegenstehende Handlungen erforderte. Dies zeigte sich vor allem in den nur marginal vorhandenen zeitlichen Ressourcen, den Kontaktbeschränkungen und einer erschwerten Kommunikation mit den Bewohner:innen.

Zusammenfassend weist die inhaltliche Auseinandersetzungen mit den Studienergebnissen darauf hin, dass sich Pflegepersonen kritisch mit der Ausgestaltung und vor allem der Einschränkung der Selbstbestimmtheit der Bewohner:innen auseinandersetzen. Die damit einhergehende Reflexionsfähigkeit bezieht sich zum einen auf das eigene (pflegerische) Handeln, zum anderen aber auch auf die Rolle der Pflegepersonen in der Pandemiebekämpfung und dem behördlichen Vorgehen des Gesundheits- und Sozialwesens. Erkennbar ist, dass Situationen, in denen die Selbstbestimmung der Bewohner:innen maßnahmenbedingt eingeschränkt wird, für Pflegepersonen moralisch herausfordernd sind. Genau an diesen Punkten versuchen sie dann durch Wohltun, also Fürsorge, Verantwortung für die betroffenen Personen zu übernehmen, um wahrgenommene Defizite zu kompensieren. Aber auch dieser Kompensationsmechanismus, der in einem Pflegealltag ohne pandemiebedingte Maßnahmen funktionieren kann, führt zu moralischen Konflikten, die durch veränderte Beziehungen zu den Bewohner:innen und einer unzufriedenstellenden Pflegequalität gekennzeichnet sind.

## Limitationen

Die Ergebnisse der vorliegenden Studie sollen vor allem dazu dienen, die durch Pflegepersonen in stationären Langzeitpflegeeinrichtungen wahrgenommenen ethischen Herausforderungen während der Covid-19-Pandemie zu dokumentieren und systematisch darzulegen. Die Reichweite der Untersuchung ist allerdings begrenzt, was die nachfolgenden Limitationen verdeutlichen:

Die Interviewstudie fokussiert ausschließlich Langzeitpflegeeinrichtungen, womit der klinische Bereich, wie auch ambulante Pflegeleistungen nicht umfasst sind.

Informationen, respektive eine Einladung zur Teilnahme an der Interviewstudie, wurden potenziellen Interviewpartner:innen erst nach institutioneller Einwilligung der Langzeitpflegeinrichtung zur Verfügung gestellt. Damit entschied die Heimleitung in einem ersten Schritt, ob Pflegepersonen Zugang zur Studie erhielten.

Des Weiteren kann davon ausgegangen werden, dass sich vor allem Pflegepersonen zur Teilnahme an der Studie gemeldet haben, die sich bereits mit dem Thema ethische Herausforderungen in der Langzeitpflege auseinandergesetzt haben, also eine Sensibilisierung für das Thema und eine damit verbundene Gesprächsbereitschaft bereits vorhanden war. Aus inhaltlicher Sicht muss zudem davon ausgegangen werden, dass die interviewten Pflegepersonen nur jene ethisch herausfordernden Situationen angesprochen haben, die sie bereits persönlich wahrgenommen bzw. erfahren haben.

Auch wenn die für die stationäre Langzeitpflege relevanten Corona-Schutzmaßnahmen bundesweit vorgegeben wurden, so liegen Alten- und Pflegeheime in der Kompetenz der Bundesländer. Diese föderale Struktur findet sich auch in unseren Daten wieder, da die vorliegende Studie ausschließlich Ergebnisse aus Interviews, die in nur einem österreichischen Bundesland (Tirol) geführt wurden, berichtet. Abschließend soll auch darauf hingewiesen werden, dass es die erhobenen Daten nicht ermöglichen, die berichteten Erfahrungen einem konkreten Zeitpunkt in der Pandemie zuzuordnen. Damit ist eine explizite zeitliche Kontextualisierung der Aussagen nicht möglich.

## Handlungsdesiderate

Die berichteten ethischen Herausforderungen in der Langzeitpflege während der Covid-19-Pandemie machen deutlich, dass Pflegepersonen mit diesen Situationen nicht allein gelassen werden sollten und ihnen Unterstützung hinsichtlich der ethischen Reflexion ihres pflegerischen Handelns angeboten werden sollte. Die in der vorliegenden Studie dokumentierten Themenbereiche zeigen auf, dass sie sich als moralische Akteure, die sich zwischen den ethischen Prinzipien Autonomie und Fürsorge bewegen, wahrnehmen und diese Position zu einem Konflikt zwischen dem individuellen Pflegeverständnis (individuelle Moral- und Wertevorstellungen) und der Realität (Begrenzung durch COVID-19-Schutzmaßnahmen) führen kann. Die Reaktion auf moralisch herausfordernde (pandemiebezogene) Situationen kann sich in Moralischem Distress (Epstein und Delgardo 2010) äußern. Moralischer Distress kann bei Pflegenden Gefühle von Frustration, Verzweiflung, Erschöpfung und Schuld hervorrufen und zu Leistungsabfall, Kontrollverlust, sinkender Arbeitsmotivation, häufiger Abwesenheit durch Erkrankung, bis letztendlich einem Austritt aus dem Beruf führen (Grønkjaer 2013; Veer 2013; Davey 2009). Morley et al. (2020b) diskutieren konkrete Beispiele von Moral Distress im Kontext der Covid-19-Pandemie und kommen zu der Schlussfolgerung, dass es essenziell ist, auf das ethische Klima der Gesundheitseinrichtung zu achten und vor allem in Ressourcen zu investieren, die moralische Unsicherheiten und Konflikte, also ethische Herausforderungen und den institutionellen Umgang damit abdecken, um den möglicherweise daraus resultierenden Moralischen Distress abzufangen.

Gegeben dem bereits vor Covid-19 unbestreitbaren Pflegenotstand ist es daher wichtig, Pflegepersonen systematisch hinsichtlich ihrer ethischen Reflexionsfähigkeit zu unterstützen (Giese 2020; Ness et al. 2021).

Das nachhaltige Etablieren von Ethikberatung in Einrichtungen der Langzeitpflege könnte dabei einen Lösungsansatz für die Komplexität ethischer Herausforderungen und moralischer Alltagskonflikte darstellen (Bockenheimer-Lucius 2007; Gschwandtner et al. 2020). Auch wenn die Einrichtung von Ethikkomitees in der Langzeitpflege – vor allem auch in Österreich – noch in den Anfängen steckt, hat die Institutionalisierung der Ethikberatung das Ziel, die Beschäftigten für ethische Fragestellungen zu sensibilisieren, pflege- und medizinethisches Wissen zu vermitteln und die Kompetenz im Umgang mit ethischen Problemen und Konflikten zu erhöhen. Dies kann sowohl durch Fort- und Weiterbildungen zu ethischen Themen (Vorträge, Workshops, Diskussionen, Veröffentlichung von Fallbeispielen, etc.) erfolgen, als auch durch die konkrete Beratung bei ethischen Problemlagen und Konflikten im Einzelfall.

Alle diese Ansätze könnten dazu genutzt werden, die (pandemiebezogenen) pflegeethischen Herausforderungen zu analysieren, daraus zu lernen und für eine nächste Pandemie vorbereitet zu sein.

## Footnotes

1 Für einen Überblick siehe https://www.aem-online.de/index.php?id=163. Zugegriffen: 1. Sept. 2021.

2 Die Stichprobengröße wurde vorab nicht festgelegt, die Auswahl der Interviewpartner:innen war offen. Der Prozess wurde im Sinne der „theoretischen Sättigung“ beendet, die gemäß Rosenthal (2011, S. 85) dann eintritt, „wenn keine Fälle mehr gefunden werden, die die Konstruktion eines neuen Typus erfordern oder die bisherigen theoretischen Verallgemeinerungen noch modifizieren könnten“.

3 https://pflege-professionell.at/at-freiheitsbeschraenkung-und-Covid-19. Zugegriffen: 1. Sept. 2021.

4 Kontaktpersonen mit Hoch-Risiko-Exposition, Klassifizierung K1 sind in Österreich definiert als Personen, die kumulativ für ≥15 min in einer Entfernung ≤2 m Kontakt von Angesicht zu Angesicht mit einem bestätigten Covid-19 Fall hatten. Personen mit Hoch-Risiko-Exposition können auf eine Klassifizierung K2 herabgestuft werden, wenn sie geimpft oder genesen sind oder wenn entsprechende Kontaktpersonen korrekt umgesetzte Maßnahmen zur Minimierung des Infektionsrisikos angewandt haben. Für weitere Details siehe www.Sozialministerium.at. Zugegriffen: 1. Sept. 2021.
